# Supplementary material for: EnzML: multi-label prediction of enzyme classes using InterPro signatures
Source: BMC Bioinformatics. 2012 Apr 25;13:61. doi: 10.1186/1471-2105-13-61 (PMC3483700; doi:10.1186/1471-2105-13-61)
Supplement: Addtional file 5 — The Java code to format the data files, evaluate and predict. The file enzml_java_code.tar.gz contains the Java code used to format database data to ARFF and XML formats, to execute cross and train-test (jackknife) evaluations and to record evaluation results to database. More information is included in the readme.txt file and the Javadoc files. The code can be used with a MySQL database. To use a different database software, other JDBC drivers might be required. [file 1471-2105-13-61-S5.gz › java_code/enzml2011/doc/uk/ac/ed/inf/taweka/mulan/class-use/MulanDataSetManager.html]

Uses of Class uk.ac.ed.inf.taweka.mulan.MulanDataSetManager


---


|  |  |  |
| --- | --- | --- |
| **Overview** \| **Package** \| **Class** \| **Use** \| **Tree** \| **Deprecated** \| **Index** \| **Help** | |  |
| PREV   NEXT | **FRAMES**    **NO FRAMES**     **All Classes** |  |


---


## **Uses of Class uk.ac.ed.inf.taweka.mulan.MulanDataSetManager**

| Packages that use MulanDataSetManager | |
| --- | --- |
| **uk.ac.ed.inf.taweka.mulan** |  |
| **uk.ac.ed.inf.taweka.mulan.attributesfilter** |  |
| **uk.ac.ed.inf.taweka.test.mulan** |  |

Uses of MulanDataSetManager in uk.ac.ed.inf.taweka.mulan

| Methods in uk.ac.ed.inf.taweka.mulan that return MulanDataSetManager | |
| --- | --- |
| `MulanDataSetManager` | `MulanArff.getDataSetManager()` |

Constructors in uk.ac.ed.inf.taweka.mulan with parameters of type MulanDataSetManager

`MulanDataSetDbLoader(MulanDataSetManager manager)`

`MulanDataSetGenerator(MulanDataSetManager mulanDataSetManager,
uk.ac.ed.inf.utils.OneToManyMap instanceAttributeMap,
uk.ac.ed.inf.utils.OneToManyMap instanceClassMap)`

Uses of MulanDataSetManager in uk.ac.ed.inf.taweka.mulan.attributesfilter

| Subclasses of MulanDataSetManager in uk.ac.ed.inf.taweka.mulan.attributesfilter | |
| --- | --- |
| `class` | `AttributesFilteredDataSetManager` |

Uses of MulanDataSetManager in uk.ac.ed.inf.taweka.test.mulan

| Methods in uk.ac.ed.inf.taweka.test.mulan that return MulanDataSetManager | |
| --- | --- |
| `static MulanDataSetManager` | `MulanDataSetManagerTest.getDataSetManager()` |
| `static MulanDataSetManager` | `MulanDataSetWriterTest.getDataSetManager(java.lang.String dataSetName)` |

---


|  |  |  |
| --- | --- | --- |
| **Overview** \| **Package** \| **Class** \| **Use** \| **Tree** \| **Deprecated** \| **Index** \| **Help** | |  |
| PREV   NEXT | **FRAMES**    **NO FRAMES**     **All Classes** |  |


---
